# ATR-hippo drives force signaling to nuclear F-actin and links mechanotransduction to neurological disorders

**DOI:** 10.1126/sciadv.adr5683

**Published:** 2025-02-14

**Authors:** Maria Chatzifrangkeskou, Tess Stanly, Delia Koennig, Luana Campos-Soares, Michael Eyres, Alexander Hasson, Alexandra Perdiou, Iolanda Vendrell, Roman Fischer, Sayoni Das, Steve Gardner, Simei Go, Ben Futcher, Ashley Newton, Paris Skourides, Francis Szele, Eric O’Neill

**Affiliations:** ^1^ Department of Oncology, University of Oxford, Oxford OX3 7DQ, UK.; ^2^ Department of Biological Sciences, University of Cyprus, P.O. Box 20537, 2109 Nicosia, Cyprus.; ^3^ Department Physiology, Anatomy and Genetics, University of Oxford, Oxford OX1 3PT, UK.; ^4^ Target Discovery Institute, Centre for Medicines Discovery, Nuffield Department of Medicine, University of Oxford, Oxford, UK.; ^5^ PrecisionLife, Bankside, Long Hanborough, Oxford OX29 8LJ, UK.

## Abstract

The mechanical environment is sensed through cell-matrix contacts with the cytoskeleton, but how signals transit the nuclear envelope to affect cell fate decisions remains unknown. Nuclear actin coordinates chromatin motility during differentiation and genome maintenance, yet it remains unclear how nuclear actin responds to mechanical force. The DNA-damage kinase ataxia telangiectasia and Rad3-related protein (ATR) translocates to the nuclear envelope to protect the nucleus during cell motility or compression. Here, we show that ATR drives nuclear actin assembly via recruitment of Filamin-A to the inner nuclear membrane through binding of the hippo pathway scaffold and ATR substrate, RASSF1A. Moreover, we demonstrate how germline RASSF1 mutation disables nuclear mechanotransduction resulting in cerebral cortex thinning and associates with common psychological traits. Thus, defective mechanical-regulated pathways may contribute to complex neurological disorders.

## Teaser

Genetic defects of mechanotransduction are identified as a psychiatric-prone syndrome.

## INTRODUCTION

The nuclear envelope (NE) has emerged as a key component of sensing mechanical forces emanating from the extracellular environment via the cytoplasm. The NE transduces signals from the cytoskeleton through the LINC (linker of nucleoskeleton and cytoskeleton) complex to the nucleus (*1*). Nucleo-cytoskeletal coupling and nuclear mechanics contributes to cell migration, development, wound healing, and immunity with mutation of NE proteins associating with adverse pathologies and accelerated aging (*2*, *3*). Ataxia telangiectasia and Rad3-related protein (ATR) is a DNA damage checkpoint kinase essential for embryonic development and loss leads to the rapid onset of a broad range of age-related phenotypes (*4*). Mutations in *ATR* are linked to Seckel syndrome characterized by severe microcephaly, growth retardation, and increased cancer risk (*5*). The N-terminal HEAT domain of ATR exhibits elastic properties, which explains how ATR can act as a mechanoresponsive kinase (*6*, *7*). ATR associates with the NE in response to mechanical stress and ensures appropriate mechanical coupling of the cytoskeleton to the NE (*8*, *9*).

As one of the first ATM/ATR kinase substrates, RASSF1A [originally PTS; (*10*)] has been subsequently validated as a *bone fide* substrate at stalled replication forks and sites of rDNA damage in nucleoli (*11*–*13*). RASSF1A is a central hippo pathway scaffold for mammalian sterile 20-like kinase 1/2 (MST1/2) and mediates large tumor suppressor kinase 1/2 (LATS1/2) regulation of the mechanosensors yes-associated protein/PDZ-binding motif (YAP/TAZ) in response to matrix stiffness (*14*, *15*). We recently showed that RASSF1A localizes to the NE where it governs nuclear actin export and SRF/MRTF-mediated transcription (*16*). Here, we uncovered a mechanically induced DNA damage response (DDR)-independent ATR-RASSF1A signaling cascade at the NE that recruits Filamin-A leading to the stabilization of nuclear actin filaments.

### ATR induces nuclear actin polymerization upon mechanical stimulation

**Fig. 1.**
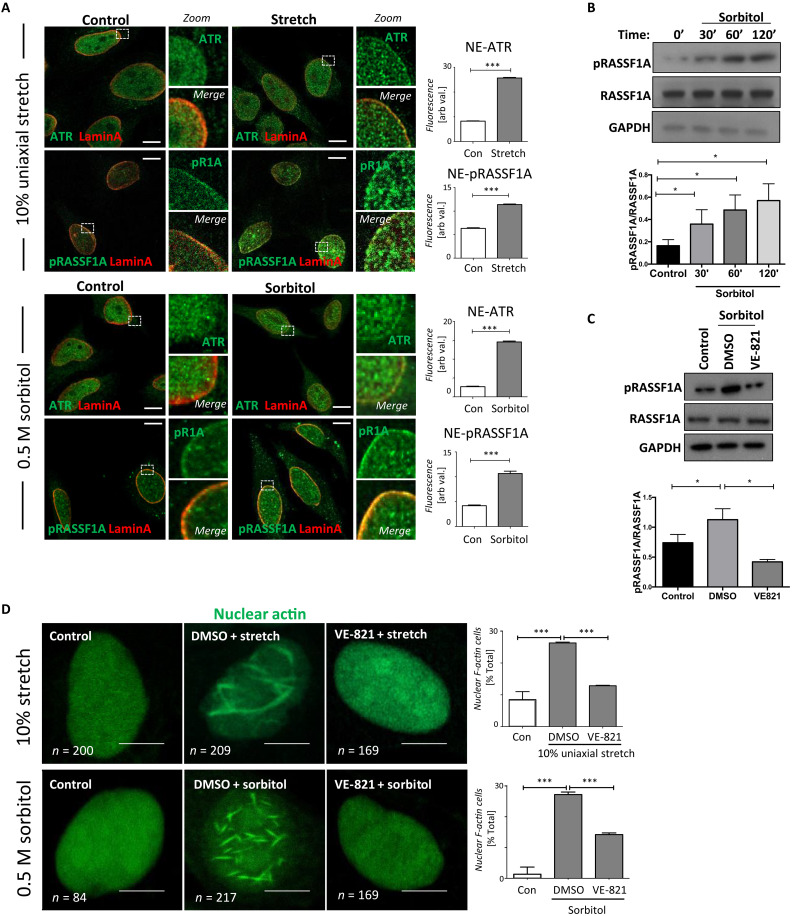
ATR-mediated phosphorylation of RASSF1A mechanical stimulation. (**A**) Representative immunofluorescence images of ATR and RASSF1A in response to 10% uniaxial cyclic mechanical stretch (top) or 0.5 M sorbitol (bottom) in HeLa cells. Scale bars, 5 μm. Graph shows quantitation of NE-ATR and NE-pRASSF1A (*n* > 100 cells). (**B**) Western blots of HeLa cells exposed to sorbitol over time and (**C**) after 30 min of sorbitol in the presence of DMSO or VE-821. (**D**) HeLa cells transfected with nuclear actin GFP chromobody under mechanical stretch or sorbitol in the presence of DMSO or VE-821. Scale bars, 5 μm. Error bars represent mean ± SEM from three experiments.

The ATR substrate pS131-RASSF1A (pRASSF1A) localizes at the perinuclear regions (*16*) and we considered that this may be driven by DNA torsional stress or cytoskeletal-mediated mechanical stress at the NE (*8*). Using direct mechanical uniaxial stretch (fig. S1A) or osmotic stress to induce mechanical force at the NE (*17*), we observe colocalization of ATR with the inner nuclear membrane (INM) protein Lamin A/C at the nuclear periphery consistent with previous reports (*8*) coincident with pRASSF1A (Fig. 1A and fig. S1, B and C). As previously reported, a pool of RASSF1A colocalizes with Lamin A/C (*16*) and remains stable under mechanical force, suggesting that increased pRASSF1A is a direct response to mechanotransduction (fig. S1D). ATR recruitment to the NE and subsequent elevation of pRASSF1A occurs within 30 min upon hyperosmotic stress using sorbitol, and this phosphorylation is lost in the presence of the specific ATR inhibitor, VE-821 (Fig. 1, B and C). RASSF1A coimmunoprecipitation (co-IP) from nuclear protein lysates also confirmed its interaction with ATR upon mechanical stimulation (fig. S1E). Notably, we did not observe accumulation of γH2AX under the experimental conditions compared to untreated samples, suggesting that the ATR/pRASSF1A mechanical signaling is independent of DNA damage (fig. S1F). In contrast, irradiation at 5 Gy for 30 min served as a control, demonstrating the expected γH2AX accumulation associated with DNA breaks. We next sought to address whether NE-ATR induced by mechanical stimulation affects export of nuclear actin via pRASSF1A (*16*). There was no discernable shift in levels of nuclear actin in the nucleus following sorbitol (fig. S1G), suggesting that NE-ATR and pRASSF1A play a distinct role to RAN-XPO6 during mechanical responses. Because nuclear actin is often difficult to visualize with phalloidin (*18*), we expressed an actin chromobody fused on its C-terminus with green fluorescent protein (GFP) tag and nuclear localization signal (NLS). We observe that mechanical force and hyperosmolarity stimulate polymerization of nuclear actin into filamentous actin (F-actin) in an ATR-dependent manner (Fig. 1D). We observed a variety of F-actin structures, ranging from small, dot-like aggregates to large, intricate networks, which may indicate the different phases of F-actin polymerization (fig. S1H). Of note, the percentage of cells with force-induced nuclear actin filaments was decreased in the absence of RASSF1A (fig. S1I). We previously showed that RASSF1A is recruited to the NE by the hippo kinase MST2 (*19*). As expected, the number of cells with nuclear actin filaments was reduced upon introduction of siRNA targeting MST2, suggesting that recruitment of RASSF1A to the INM is required (fig. S1J). Because total nuclear actin levels remained static, we infer that nuclear F-actin filaments induced by mechanical force likely arise from a preexisting pool of monomeric nuclear G-actin.

### Filamin-A mediates nuclear F-actin

**Fig. 2.**
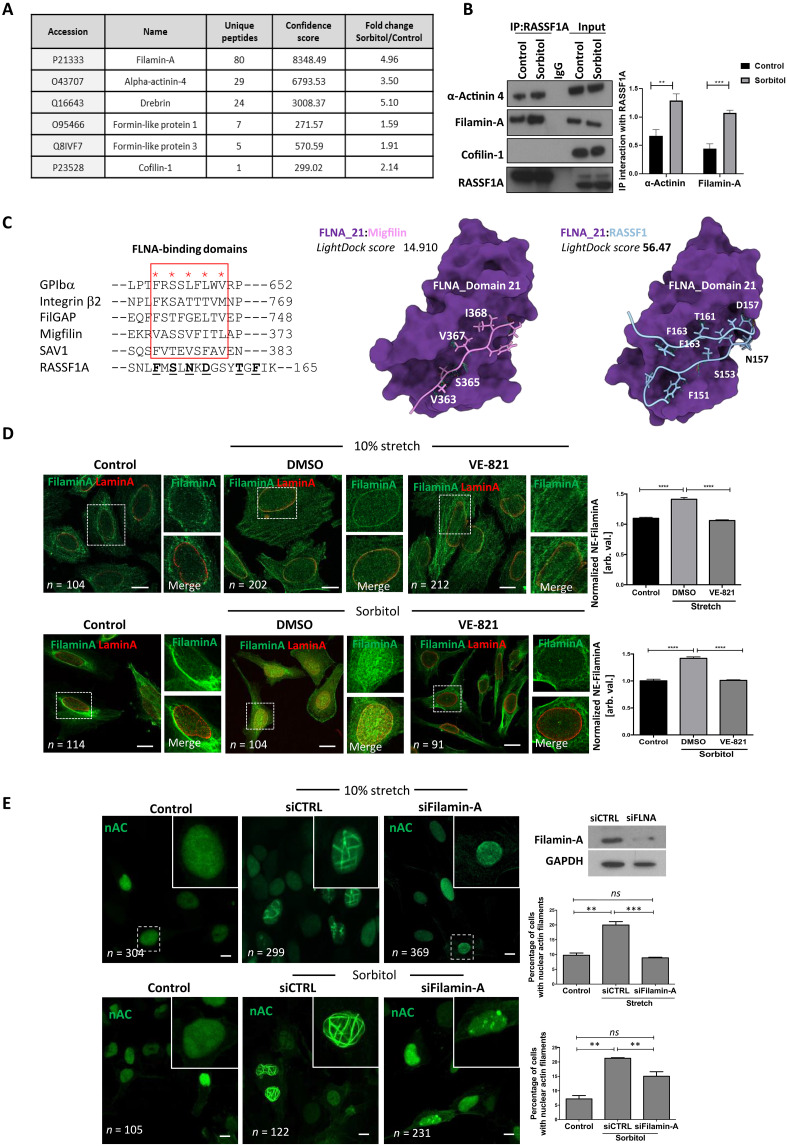
Filamin-A mediates nuclear actin formation upon mechanical stimulation. (**A**) LC-MS/MS hits identified in RASSF1A IP. The table includes the MASCOT score and number of unique peptides after applying a 20 ion cutoff and 1% FDR. (**B**) Immunoprecipitation and Western blot of RASSF1A with α-actinin 4 and Filamin-A with bars representing quantitation of bands. (**C**) A comparison between binding sites of Filamin-A–binding partners and the FLNA-binding sequence of RASSF1. Docking of FLNA-binding domains of Migfilin (pink) and RASSF1 (blue) with associated LightDock scores. (**D**) Immunofluorescence images of Filamin-A with Lamin A/C in HeLa cells upon mechanical stretch (top) and sorbitol (bottom) cells in the presence of DMSO or VE-821. Scale bars, 5 μm. (**E**) Immunofluorescence images of nuclear actin in HeLa cells upon mechanical stimulation (top) and sorbitol treatment (bottom) transfected with siRNA control (siCTRL) or targeting Filamin-A (siFilamin-A); siRNA efficiency shown in Western blot and bars indicate quantification. Scale bars, 5 μm. Error bars represent mean ± SEM from at least three independent experiments.

To identify candidate mediators of nuclear actin polymerization upon mechanical force, we performed mass spectrometry of endogenous RASSF1A immunoprecipitates from control versus sorbitol-exposed HeLa cells. A number of actin regulatory proteins were identified with specific enrichment of Filamin-A and α-actinin 4 (ACTN4) under hyperosmotic conditions (fivefold and threefold, respectively, Fig. 2A and tables S1 and S2). ACTN4 has previously been implicated in nuclear F-actin–mediated mobility of postmitotic nuclei (*19*) and Filamin-A localizes to the nucleolus where rDNA transcription is regulated by RASSF1A and nuclear F-actin (*13*, *18*). Increased interaction of both Filamin-A and ACTN4 with RASSF1A upon mechanical stress was confirmed via co-IP (Fig. 2B). We identified a typical Filamin-A-binding motif in RASSF1A in the central RA-domain downstream of S131, predicted by the alignment of the binding sites of known Filamin-A–binding proteins (Fig. 2C, left). Taking advantage of known crystal structures of mechanoresponsive domain 21 in Filamin-A that are exposed under molecular tension (*20*), we questioned whether the proposed affinity of RASSF1A-binding site peptide could be determined via the LightDock server (*21*). In line with the results above, RASSF1A is predicted to dock in a similar manner to the known substrate Migfilin (Fig. 2C, right). To examine whether Filamin-A association also results in its recruitment to the NE, we imaged cells following mechanical stress and observed robust colocalization of Filamin-A with Lamin A/C (Fig. 2D and fig. S2A). Notably, ACTN4 did not associate with the NE, implying a potential role during mitosis and cytokinesis rather than interphase cells (fig. S2B). Furthermore, knockdown of RASSF1A (fig. S2C) or inhibition of ATR abrogated mechanical force–induced Filamin-A recruitment to the NE (Fig. 2D). Overall, our data demonstrate that Filamin-A recruitment to the lamina under mechanical force is dependent on ATR-mediated RASSF1A phosphorylation. As a constituent of the cytoskeleton, Filamin-A is mostly localized in the cytoplasm of unstressed cells but is also observed at the nucleolus and in DNA repair foci (*22*, *23*). To distinguish whether Filamin-A associates with inner or outer nuclear membrane (INM versus ONM), we compared permeabilization of total membranes using Triton X-100 versus digitonin, which selectively permeabilizes the plasma membrane to leave the INM intact, and find mechanical force induces Filamin-A accumulated at the INM (fig. S2D). As this supports a potential role in nuclear F-actin assembly, we restricted the expression of Filamin-A and observed a reduction in the number of cells exhibiting nuclear F-actin under mechanical force (Fig. 2E).

### Genetic disruption of ATR-RASSF1A-actin leads to neurological effects

**Fig. 3.**
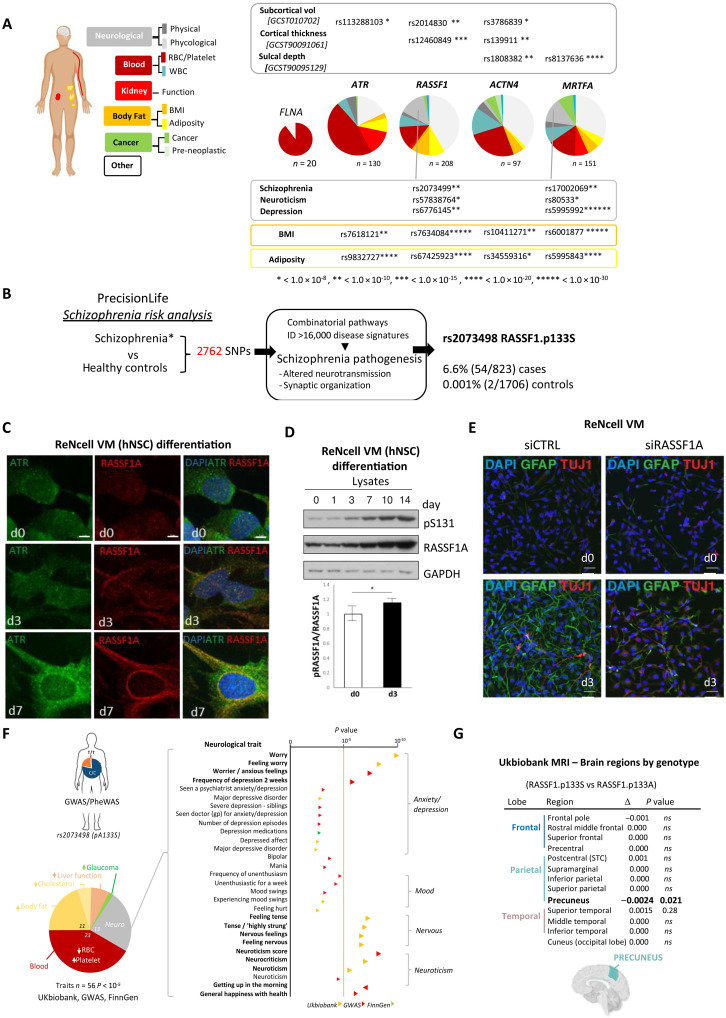
ATR-RASSF1A affects neuronal differentiation and neurological disorders. (**A**) Analysis of opentargets.org for pathway gene SNPs associated with the indicated phenotypic traits. (**B**) SNP and disease signature combinatorial pathways analysis performed by PrecisionLife using data from UK Biobank. (**C**) Representative images of ReNcell VM cell differentiation on indicated days after EGF/FGF withdrawal, stained with indicated antibodies; scale bars, 5 μm or (**D**) lysates subjected to Western blot and intensity at day 3 quantified. (**E**) Differentiating VM cells nucleofected with siRNA control (CTRL) or targeting RASSF1 (siRASSF1) at day 3 and stained for markers of glial cells (GFAP, green) and neurons (TUJI, red). Scale bars, 50 μm. (**F**) Analysis of opentargets.org for rs2073498 association with indicated phenotypic traits. (**G**) ENIGMA-cortical thickness variations associated with rs2073498.

Mutations in Filamin-A are notably associated with X-linked hereditary periventricular heterotopia (*24*) but more recently with subtle phenotypes of the connective tissue syndrome Ehlers-Danlos hypermotility subtype (*25*). This points to Filamin-A defects affecting neuronal migration, mechanosensation, and extracellular matrix (ECM) deposition. To determine the physiological relevance of NE Filamin-A and nuclear F-actin, we explored genetic associations of ATR, RASSF1, Filamin-A, ACTN4, and nuclear g-actin-responsive MRTFA (regulated by RASSF1A) from large-scale human biobanks [FinnGen, UKBiobank, genome-wide association studies (GWAS); www.opentargets.org]. Despite the severe impact of Filamin-A mutations, genetic variance has not yet been observed to critically affect health. However, ATR, RASSF1, ACTN4, and MRTFA all harbor single-nucleotide polymorphisms (SNPs) that consistently associate with variations grouped into neurological, body fat, and cancer (Fig. 3A). Specifically, we find consistent associations with body mass index (BMI) and adiposity, which may be attributable to regulation of adipose differentiation, via mechanoresponsive YAP/TAZ localization that are governed by nuclear F-actin (*26*, *27*). We noted a notable association of multiple SNPs in RASSF1 and MRTFA with psychological traits schizophrenia, neuroticism, and depression. Notably, numerous shared genetic loci between BMI and major psychiatric disorders have been reported, suggesting potential shared pathophysiological mechanistic routes (*28*). Variations in cerebral cortex thickness are commonly associated with psychiatric disorders, and we find regional variations of the cerebral cortex, subcortical regions, and sulcal depth across ATR, RASSF1, ACTN4, and MRTF, suggesting a potential mechanistic link to mechanical force signaling described above (Fig. 3A).

We next sought to independently validate the association of mechanical force signaling at the NE with psychiatric traits and took advantage of the PrecisionLife platform that integrates SNPs with disease signatures (combinations of SNPs) associated with pathogenesis (*29*) providing a comprehensive schizophrenia risk analysis (Fig. 3B) performed using genomic data from UKbiobank (*30*). From this, we identified noncoding SNPs in RASSF1 such as rs2073499, and the nonsynonymous rs2073498 (RASSF1A-A133S), which encodes for a Ser at position 133 of RASSF1A that restricts ATR phosphorylation at Ser^131^ (*31*). The minor allele associates with neuroticism (*32*) and cognitive decline in early Alzheimer’s (*33*), and we find increased risk of schizophrenia as prevalent in 6.6% of cases but almost absent from controls (Fig. 3B).

### ATR-RASSF1A regulates neuronal differentiation

Previously, we found that RASSF1A promotes differentiation of embryonic stem cells (ESCs) toward a neuronal lineage via PAX3 and SOX1 (*34*). Thus, rs2073498 association with schizophrenia may suggest that ATR-RASSF1A mechanotransduction plays a role in neuronal differentiation. We next followed H9 human embryonic stem cells (hESC) during maintenance (mTeSR1) and differentiation (StemDiff) toward all three germ layers, ectoderm, endoderm, and mesoderm, and observed increased pRASSF1A expression increases during differentiation (fig. S3A,B). Notably, pRASSF1A is detectable in hESC cells and suppressed by ATRi not ATM or TGFbi, suggesting that ATR-RASSF1A plays a role during differentiation (fig. S3C). To determine whether this supports neuronal development, we differentiated hESCs down a neuroectodermal lineage to PAX6^+ve^, NESTIN^+ve^ neuronal progenitor cells (fig. S3D). We see the initial expression of RASSF1A in neuroectodermal cells increase as cells become neural progenitors, with an enrichment in the nuclear compartment (fig. S3D). Ventromedial (VM) human neural progenitor cells (hNSCs) present a later stage in neurogenesis than ESC and can differentiate into mature neural cells 7 to 14 days after withdrawal of EGF and FGF (*35*). Within 3 days, both ATR and RASSF1A accumulate at the NE coincident with increased pS131-RASSF1A and is followed by increasing levels of RASSF1A stability and higher pRASSF1A (Fig. 3, C and D, and fig. S3E). VM cells readily differentiate into neurons, astrocytes, and oligodendrocytes (*35*). Therefore, to determine whether RASSF1A affects differentiation, we monitored the number of GFAP^+ve^ (glial) and TUJI^+ve^ (Tubulin βIII, neuron) cells, respectively, and restricted RASSF1A expression with siRNA. We see increased numbers of GFAP^+ve^ with a few TUJI^+ve^ cells within 3 days of differentiation and an almost complete loss of both GFAP and Tubulin βIII expression in the absence of RASSF1A expression (Fig. 3E and fig. S4A). Notably, restriction of ATR activity or RASSF1 expression in differentiating VM cells appears to specifically limit levels of MAP2, a microtubule stabilizing neuronal differentiation marker (fig. S4, B to D), which may explain why Tubulin βIII expression remains unaffected but higher-order stabilization is not readily visualized in cells (TUJI, Fig. 3E). There is evidence for reduced MAP2 expression or aberrant regulation in schizophrenia and psychiatric disorders (*36*), and MAP2 loss may indirectly affect GFAP^+ve^ glia (*37*). Conversely, overexpression of RASSF1A appears to accelerate GFAP accumulation before differentiation and results in more stable GFAP^+ve^ processes by d3 (fig. S4E).

As ATR-RASSF1A appears to be involved in glial cell function and neuronal differentiation, we analyzed rs2073498 as above (Fig. 3A) and find similar associations with body fat and neurological traits including association with neuroticism, nervousness, and anxiety with an accumulative trend toward significance for low mood and depression (Fig. 3F). We took advantage of Enigma magnetic resonance imaging (MRI) brain data from genetic cohorts to assess whether regional cortex brain volumes vary in rs2073498 carriers that may explain trait association (*38*). ENIGMA suggests a reduced precuneus volume in minor allele carriers (RASSF1A-133S) (Fig. 3G and table S2). This region of the parietal lobe contributes to the “default mode network,” which connects to the cerebellum via Purkinje neurons (*39*) and is hypothesized to be relevant to disorders including Alzheimer’s, autism, schizophrenia, major depressive disorder, chronic pain, and posttraumatic stress disorder (*40*). Moreover, as cortical thickness is also associated with neurological disorders (*41*), we potentially can link genetic traits ATR-RASSF1A-actin to disruption of mechanotransduction, nuclear F-actin and neuronal plasticity, cortex depth, and psychiatric disorders. GWAS studies require genetic associations to be entirely independent; therefore, only strong associations reach the stringent significance threshold (*P* = 1.0 × 10^−8^), with several important traits failing identification. Our data on pRASSF1 identified an impactful coding polymorphism, which together with results from ENIGMA and opentargets, prompted us to validate the potentially pathogenic impact of rs2073498 in vivo.

### *Rassf1A*^*A133S/A133S*^ mice phenocopy impact of rs2073498 on ATR-RASSF1A-actin

**Fig. 4.**
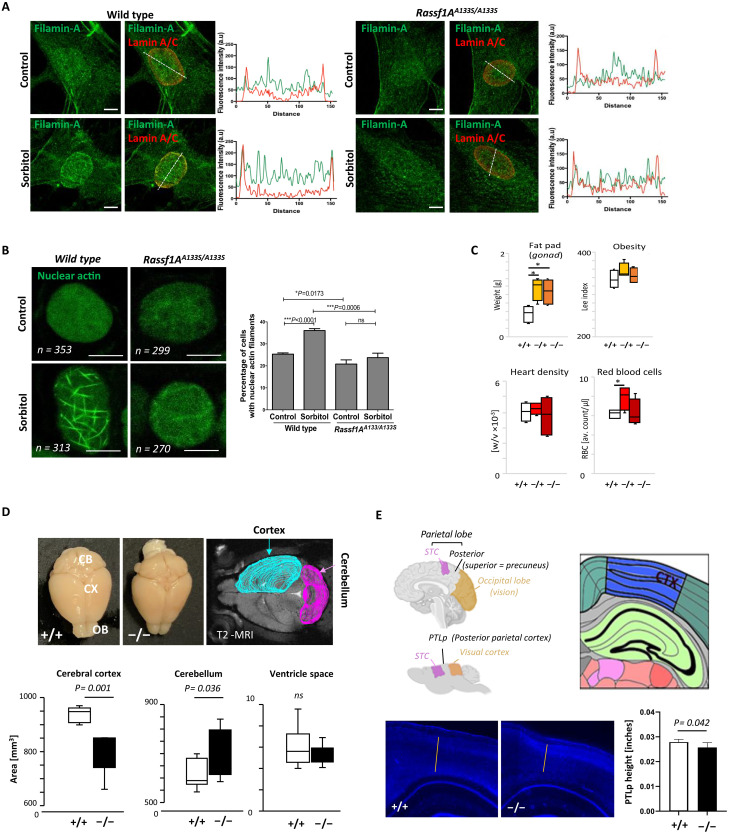
Failure to respond to force in *Rassf1A*^*A133S/A133S*^ MEFs. (**A**) Representative immunofluorescence images and fluorescence intensity profiles of Filamin-A in MEFs from WT and *Rassf1A*^*A133S/A133S*^ mice in the presence or absence of 0.5 M sorbitol. Scale bars, 5 μm. (**B**) Confocal images of nuclear actin (chromobody) in WT and *Rassf1A*^*A133S/A133S*^ MEFs in the presence or absence of 0.5 M sorbitol. Scale bars, 5 μm. Quantification of nuclear actin filament increases in WT and Rassf1AA133S/A133S MEFs in response to 0.5 M sorbitol or uniaxial stretch. Error bars represent mean ± SEM from three experiments. (**C**) Characterization of *Rassf1A*^*WT/WT*^ (+/+), *Rassf1AWT/A133S* (−/+), and *Rassf1AA133S/A133S* (−/−) for general physiological parameters; adiposity (gonadal fat pad weight), obesity (Lee index), heart density (weight × volume), and red blood cell count, *n* = 6/group. (**D**) ImageJ segmentations for cerebral cortex MRI indicating regions selected for volume (ImageJ), with bars representing an area of *n* = 6 mice per group). (**E**) Representation of somatosensory region of the cerebral cortex containing the precuneus (human) and representative region, PTLp (mice). Sections stained with DAPI, and cell depth estimated as cortical height. Error bars represent mean ± SD.

RASSF1 rs2073498 major allele is developmentally conserved in mice, which allowed us to generate *Rassf1A-A133S* mice (fig. S5, A to C). Mice are born without obvious developmental defects but develop spontaneous cystic pathologies in the genital tract, kidneys, and pancreas and develop cancer cells with age that only moderately affects overall survival (fig. S5, D and E). Of note, A133S substitution does not affect the association of RASSF1A with the NE or its expression levels in both the nucleus and cytoplasm of mouse embryonic fibroblasts (MEFs) (fig. S5F). As ATR-mediated pS131-RASSF1A is restricted by A133S substitution, we next determined whether MEFs from *Rassf1A*^*A133S/A133S*^ mice have a reduced ability to recruit Filamin-A to the NE. We observe a clear recruitment under mechanical force in MEFs from littermate controls that is lost in *Rassf1A*^*A133S/A133S*^ MEFs (Fig. 4C). Notably, whereas controls’ MEFs readily establish stable nuclear F-actin in response to sorbitol or force, *RASSF1A*^*A133S/A133S*^ MEFs also failed to do so (Fig. 4D and fig. S5G). These results collectively indicate that NE-ATR phosphorylates NE-RASSF1A and mediates a nuclear response to mechanical stress through the nuclear actin filament formation from existing nuclear actin pools.

**Fig. 5.**
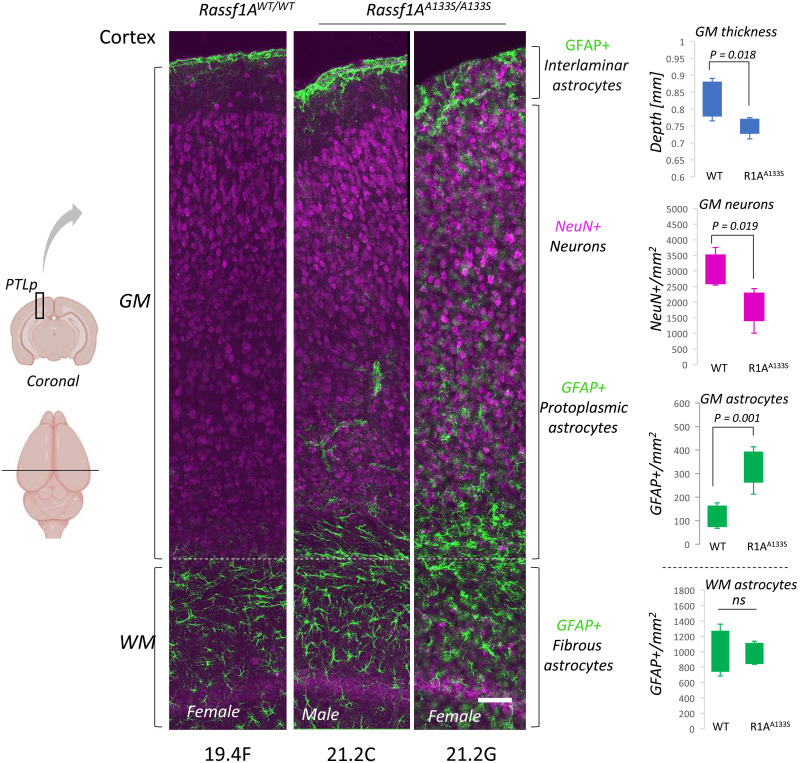
Pathological impact of failure to respond to force in Rassf1A^A133S/A133S^ mice. Serial coronal sections of mice brains, 30 μm, (1.75 to 2.8 mm posterior to bregma) were taken for immunostaining. Maximum intensity projections of confocal images, 200 μm sections of the brain (black box), highlighting the PTLp regions were analyzed for GFAP (green) and NeuN (magenta). GM depth was defined from the region where NeuN staining initiates in the base of the cortex (white dotted line) to the top of cortex. WM for this instance was simply defined as a selected NeuN-ve depth below GM. Unpaired Student’s t test; scale bar, 100 μm.

We next monitored *Rassf1A*^*A133S*^ mice for phenotypes that were associated with rs2073498 in humans and found that there is an increased association with gonadal fat in hetero- and homozygote mice that correlates with adiposity and BMI traits observed in human cohorts above (Fig. 5A). To interrogate correlation with brain volumes, we performed MRI of wild-type (WT) and Rassf1A^A133S/A133S^ mice. Analysis of the cerebral cortex size showed a notable reduction in *Rassf1A*^**A133S*/A133S*^ compared to that of WT mice with potential collateral increase in the cerebellum compared to overall ventricle space (Fig. 5B and fig. S6A), which is independent of age and gender (fig. S6B). To examine whether variations are evident in specific regions correlating with the precuneus, we also took sections from equivalent areas between the somatosensory cortex (STC, pink) and the occipital lobe/visual cortex (gold), the posterior parietal cortex (PTLp, Fig. 5C). We find that cortical depth of the entire cortex (blue) in coronal sections stained with 4′,6-diamidino-2-phenylindole (DAPI) shows a marked reduction in PTLp height between WT and *Rassf1A*^*A133S/A133S*^ mice (Fig. 5C). These results collectively indicate that the decrease of the cerebral cortex stemming from the loss of RASSF1A may be due to its inability to interact with Filamin-A. To better understand the consequence of rs2073498 and perturbing nuclear F-actin on the cortex, we stained the PTLp for the major cell markers of glial cells (GFAP) and neurons (NeuN). We observe substantial alteration in the gray matter (GM) depth within the cortex, which correlates with a reduced number of NeuN+ neurons in this region of *Rassf1A*^*A133S/A133S*^ mice. We see a concomitant increase and dispersion of differentiated GFAP+ cells throughout the GM of cortex, while the white matter (WM) astrocytes show little change (Fig. 5D and fig. S7). The observed variations in cortex GM depth were again independent of gender/age and may suggest either skewed differentiation of radial glial cells during development or inflammatory activation of glia and destruction of neurons in adults. Together, our data support that ATR-hippo signaling is an important mediator of nuclear F-actin and the impact of an rs2073498 has a physiological impact supported by human genetic association with psychiatric disorders.

## DISCUSSION

Phenotypic ataxia associated with ATR loss has been described as a consequence of stem cell depletion resulting in progressive loss of Purkinje neurons in the cerebellum (*5*). Regulatory functions for ATR beyond DNA damage have been implicated in the formation of dendritic spines, synaptic firing, synaptic remodeling, and neuronal plasticity (*42*, *43*). Notably, ATR facilitates nuclear F-actin formation to mobilize chromatin before DNA repair (*44*) and promotes axon regeneration in a DDR-independent manner (*43*). ATR has emerged as a central mechanosenor of the NE, required for nuclear responses to mechanical force and ATR-defective cells fail to maintain cyto-nuclear force coupling (*9*). ATR inactivation, e.g., in Seckel syndrome, results in not only microcephaly and ataxia but also behavioral changes in mice (*5*, *42*). Of note, microcephaly was also observed during Zika viral infection that caused hypermethylation and inactivation of RASSF1 (*45*). Less penetrant SNPs in DNA repair genes have been associated with cognitive impairment, schizophrenia and depression, considered both as a failure of oxidative stress resolution in neurons and a consequence of defective neuronal enhancer regulation (*46*–*48*). Similarly, psychological traits are enriched with loss of cell-cell adhesion and plasma-membrane adhesion function (*47*, *49*), further implicating sensing of the mechanical environment in neuropsychiatric disorders. Thus, ATR mechanotransduction is likely to play a major role in protecting mental health. More work is needed to assess the genetic risk, precise neurobiology, and potential phenotypes in mice, but our hippo-related mechanism presented here would contribute to ATR function.

Uniaxial stretch induces changes in cell shape, alignment of cytoskeletal elements, and nuclear deformation, reflecting the mechanical forces encountered by cells in tissues such as in response to growth, wound healing, or mechanical strain (*50*, *51*). Osmotic stress is a potent inducer of mechanotransduction at cell membranes, leading to substantial changes in cell volume due to water influx or efflux, and mechanical interactions with the ECM (*17*). When cells are subjected to osmotic shock, alterations in cell volume and cortical tension result in the deformation of the ECM, which affects the mechanical forces between cells and the ECM (*52*, *53*). These two distinct types of mechanical forces activate unique mechanosensory pathways, with uniaxial stretch being more relevant to tissue integrity and cellular alignment under mechanical strain, while osmotic stress influences cellular volume regulation and ECM interaction.

Formation of transient nuclear F-actin is induced by integrin-mediated cell adhesion, cell spreading, mitotic exit of cells and a variety of cell stress cues (*54*–*56*). As in the cytoplasm, actin-binding proteins (ABPs) are also found in the nucleus (*57*) and induction of nuclear F-actin following cell spreading family involves mDia1 and mDia2, whereas Formins and Spire-1/2 mediate nuclear actin assembly in response to DNA damage (*56*). Recent studies in T cells show that T-cell receptor (TCR) engagement stimulates nuclear actin polymerization via mDia with N-WASP and Arp2/3 complex also playing a role (*58*). The actin cytoskeleton has been implicated as a convergent mechanism for mental health disorders, through structural failures in synapses (*59*). However, as defects in ECM can also drive abnormal neural wiring leading to behavior changes and triggering stress or depression can induce ECM changes in the brain, the role for mechanical force signaling is likely to be important for neuronal phenotypic plasticity (*60*). Actin filaments can be organized into cross-linked networks or parallel bundles by filamin resulting in increased stability (*61*) or can drive liquid phase separation via cross-linking (*62*), which may support the formin disheveled associated activator of morphogenesis 2 (DAAM2)-mediated transcriptional-droplet control of androgen receptor (*63*). Notably, deleterious mutations in Filamin-A that impair actin binding result in the defective migration of neurons in the cerebral cortex that causes periventricular nodular heterotopia and is linked to neuropsychiatric disease (*24*, *64*). Further work is required to elucidate the precise consequences of mechanoresponsive nuclear F-actin on genome regulation, cell fate and migration as it is clear that deleterious mutations have a strong impact. Exactly how genetic traits present milder phenotypes or even combine to trigger pathologies remains to be understood, but our work suggests a direction for study in mental health disorders. In addition to its well-documented role in cancer and other disease contexts, nuclear F-actin’s involvement in cellular movement during confined spaces in early development presents an intriguing area for further exploration. During embryonic development, cells frequently navigate through narrow extracellular environments, which poses major mechanical challenges (*65*). Our findings suggest that the ATR-hippo axis–driven nuclear F-actin response might play a crucial role in maintaining nuclear integrity and functionality under such conditions. Specifically, the ability of nuclear F-actin to stabilize nuclear architecture and facilitate mechanotransduction could be critical for cellular adaptation during migration through confined spaces (*66*, *67*). Several studies have highlighted similar mechanisms in cancer metastasis, where cells encounter mechanical constraints that require dynamic changes in nuclear structure (*68*, *69*). By drawing parallels to these processes, we propose that nuclear F-actin may serve a comparable function in developmental contexts, where the mechanical demands of confined movement are also prominent. This perspective not only underscores the fundamental biological relevance of our findings but also opens previously unknown avenues for investigating how mechanotransduction pathways influence developmental cell behaviors.

The hippo pathway is a mechanoresponsive signal cascade that modulates transcription via YAP/TAZ in a nuclear F-actin–dependent manner (*27*) and is implicated in schizophrenia, bipolar disorder, and autism (*70*, *71*); although unknown, it may involve mechanisms similar to memory formation (*72*). RASSF1A directly activates central hippo kinases MST1/2, responds to ECM signaling, and regulates nuclear actin (*15*, *16*). Notably, MST1/2 influences major psychiatric disorders (*73*–*75*) and RASSF1 expression is associated with bipolar disorder and postpartum depression [European patent EP3819387A1, (*76*)]. Together, our study displays evidence for a mechanosensory pathway at the NE that stimulates the formation of nuclear F-actin. As F-actin is increasingly appreciated to play fundamental roles in nuclear architecture, DNA regulation, and cell fate decisions, ATR-RASSF1A is likely to have a wide impact in mechanobiology. As rs2073498 presents a common mutation in the human population, this is a potential low-penetrant syndrome that manifests chronic failures in mechanotransduction, including neurological neuroticism and schizophrenia, and corroborated by Rassf1A^A133S^ mice that display polycystic kidney disease, cancer development, fat deposition, and neurological features.

## MATERIALS AND METHODS

### Tissue culture and cell treatments

HeLa cells were cultured in complete Dulbecco’s minimum essential medium supplemented with 10% fetal bovine serum in 5% CO_2_ and 20% O_2_ at 37°C. Hyperosmotic stimulation was induced by the addition of 0.6 M sorbitol (Sigma) to the growth medium. An inhibitor of ATR kinase activity (VE-821) was used at a concentration of 10 μM. Irradiation was carried out using a Gamma Service GSRD1 irradiator containing a Cs137 source. Cells were exposed in 5 Gy for 30 min and then lysates were collected as described below.

hESCs, H9 (#WA09, WiCell), were grown on Matrigel (Corning)–coated plates in mTeSR1 media with ROCK inhibitor (Y27632, Tocris). To differentiate to ectodermal, endodermal, or mesodermal cells, they were grown on the STEMdiff Trilineage Differentiation Kit (STEMCELL Technologies), and after 7 days to generate neuroectodermal cells, the medium was supplemented with 10 μM Y-27632. Human fetal neural progenitor cells, ReNcell VM (Merck Millipore), were cultured on Laminin-coated plates in ReNcell NSC medium (Merck Millipore) containing FGF2 and EGF. To differentiate these cells, the growth factors were withdrawn from culture. H9 and ReNcell were treated with inhibitor VE-821 at 10 μM and ATM inhibitor KU55933 at 1 μM.

### siRNA oligonucleotides/plasmids

RNA interference was carried out with 100 nM siRNA for 48 hours using Lipofectamine 2000 (Invitrogen) or Lipofectamine 3000 (Invitrogen) for H9 cells according to the manufacturer’s instructions. siRNA against Luciferase with the sequence GCCAUUCUAUCCUCUAGAGGAUG was used as control. siRNA against RASSF1A was previously used (*16*). siRNA against ATR (sc-29763) was purchased from Santa Cruz Biotechnology. siRNA against Filamin-A was purchased from Origene (#SR301624). NLS-GFP-actin chromobody was purchased from Chromotek and was transfected to cells using Lipofectamine 2000 for 24 hours. pcDNA3 and FLAG-RASSF1A as previously described in Hamilton *et al.* (*11*) were used. ReNCell hNSCs were nucleofected (Nucleofector 2b device, LONZA) with siRNA or plasmid according to the manufacturer’s instructions.

### Antibodies

The following antibodies were used in this study: RASSF1A (1:200 in IF and 1:1000 in WB, Atlas, #HPA040735), Lamin A/C (1:2000 in IF and WB, Cell Signaling Technology, #4777), Lamin A/C (1:1000 in IF, Proteintech, #10298-1-AP), GAPDH (1:2000 in WB, Cell Signaling Technology, #97166), GAPDH (1:3000 in WB, Santa Cruz Biotechnology, #sc-32233), ATR (1:200 in IF and 1:1000 in WB, Cell Signaling Technology, #13934), Filamin-A (1:100 in IF and 1:1000 in WB, Cell Signaling Technology, #4762), α-actinin 4 (1:500 in IF and 1:1000 in WB, Santa Cruz Biotechnology, #sc-390205), actin (1:1000 in WB, Santa Cruz Biotechnology, #sc-47778), and pS131-RASSF1A custom-made (1:50 in IF and 1:300 in WB) (*11*). Pax6 (1:1000 BioLegend 901301), Nanog (1:1000 Cell Signaling Technology 4903), Oct4 (1:1000 Cell Signaling Technology 2840), GFAP (1:400 in IF Invitrogen 130300), Nestin (1:1000 Santa Cruz sc-21248), TUJ1 (1:400 for IF and 1:1000 for WB BioLegend 801201), MAP2 (Cell Signaling Technology 8707S), CHK1 (Santa Cruz sc-8408), CHK1-pS345 (1:500 Cell Signaling Technology 2341), actin (60008-1-1g, Proteintech), NeuN (1:300, Merck Millipore, MAB377), and GFAP (1:200, Sigma, G9269).

### Protein extraction and immunoblotting

Cytosolic and nuclear fractions were prepared using the NE-PER Nuclear and Cytosolic Extraction Reagents (Thermo Fisher Scientific) according to the manufacturer’s instructions. Extracts were analyzed by SDS–polyacrylamide gel electrophoresis (SDS-PAGE) using 4 to 12% bis-tris Nu-PAGE gels (Invitrogen) and transferred onto polyvinylidene difluoride (PVDF) membranes (Millipore). The membranes were washed in PBS containing 1% Tween 20 (PBS-T), blocked in 5% bovine serum albumin (BSA) in PBS-T for 1 hour, and then incubated with the primary antibody overnight at 4°C. The membranes were incubated with horseradish peroxidase (HRP)–conjugated secondary antibodies for 1 hour at room temperature and exposed to x-ray film (Kodak) or imaged on a ChemiDoc Touch imager (Bio-Rad). ImageJ software (NIH) was used for the quantification of the bands. All bands were normalized against the loading controls. H9 and ReNCell extracts were run on NuPage Novex 10% bis-tris gel and transferred onto a PVDF membrane and blotted as above.

### Cell nuclear extraction

HeLa cells were first lysed in fractionation buffer (20 mM Hepes, pH 7.4, 10 mM KCl, 2 mM MgCl_2_, 1 mM EDTA, and 1 mM EGTA) supplemented with 1 mM DTT and protease inhibitors and incubated on ice for 15 min. Afterward, the cells were homogenized by 10 passages through a 25-G needle using a 2-ml syringe and incubated on ice for 30 min. Nuclei were separated by centrifugation at 720*g* at room temperature for 10 min. The nuclear pellet was resuspended and lysed fractionation buffer.

### Immunofluorescence

HeLa cells were grown on coverslips, washed with PBS, and fixed with MeOH at 20°C for 20 min. Where indicated, cells were instead fixed in 4% paraformaldehyde (PFA) and permeabilized either with 0.5% Triton X-100 at room temperature for 5 min or with digitonin (40 μg/ml) on ice for 2 min. Coverslips were then incubated with primary antibody for 2 hours in PBS with 3% BSA at room temperature. Cells were washed with PBS and incubated with secondary anti-rabbit and/or anti-mouse or anti-rat immunoglobulin G (IgG) conjugated with Alexa Flour 488 or Alexa Flour 568 (Molecular Probes) for 1 hour at room temperature. Samples were washed and mounted on coverslips with mounting medium containing DAPI (Invitrogen). Cells were analyzed using an LSM780 OR LSM900 (Carl Zeiss Microscopy) confocal microscope. Maximum intensity projections of the images were generated using Fiji/ImageJ. H9 and ReNCell VM cells were grown on either Matrigel- or Laminin-coated coverslips as described previously. The cells were allowed to attach for at least 48 hours before fixing in 5% PFA and permeabilization with 0.3% Triton X-100 for 5 min. Cells were washed in PBS and blocked with 3% BSA for 1 to 2 hours at room temperature (RT). Primary antibodies were made up in 3% BSA and incubated overnight at 4°C. Cells were washed in PBS and counterstained with secondary antibodies for 2 hours at RT. Images were taken using LSM780 and processed with the ZEN software.

Coronal sections (30 μm) of mice brains (1.75 to 2.8 mm posterior to bregma) were incubated in 50 mM glycine for 15 min and washed in PBS. Nonspecific binding was blocked using 10% donkey serum and 0.5% Triton X-100 for 1 hour. Sections were incubated with primary antibodies overnight at 4°C, washed, and incubated with secondary antibodies, Alexa Flour 488, or Alexa Flour 568 (Molecular Probes) for 1 hour. DAPI staining (1:1000) was performed for 15 min. After washing in phosphate buffer (pH 7.4), sections were mounted on Superfrost Plus slides with FluorSave mounting media.

Confocal z-stacks were acquired using Andor Dragonfly (Oxford Instruments), 63× lens with a numerical aperture of 1.4. Images were stitched using Imaris (Oxford Instruments). Analysis was performed on Fiji/ImageJ. Maximum intensity projections of the ~200-μm sections of the brain were generated. From these images, GM thickness was defined from the region where NeuN staining initiates in the base of the cortex to the top of cortex. WM was defined as regions from where cortical NeuN staining ends. GM NeuN^+^ neurons and GFAP^+^ astrocytes were manually counted using Fiji/ImageJ.

### Immunoprecipitation

Cells were resuspended in lysis buffer [50 mM tris-HCl, pH 7.5, 0.15 M NaCl, 1 mM EDTA, 1% NP-40, 1 mM Na_3_VO_4_, complete proteinase inhibitor cocktail (Roche)]. Resuspended cells were lysed by passing through a 25-G needle 10 times. Cell lysates were incubated with 20 μl of protein G Dynabeads (Invitrogen) and 2 μg of the indicated antibodies for 2 hours at 4°C. Pelleted beads were collected in sample buffer NuPAGE LDS (Thermo Fisher Scientific) with 200 mM DTT and subjected to SDS-PAGE and immunoblotting using indicated antibodies. For immunoprecipitation of endogenous RASSF1A, 500 μg of total protein was incubated with 2 μg of the anti-RASSF1A overnight at 4°C with gentle rotation. For nuclear specific immunoprecipitation assays, nuclear extracts were incubated with anti-RASSF1A antibody overnight at 4°C. Following this, 20 μl of protein G Dynabeads (Invitrogen) was added to the mixture, and the incubation was continued for an additional 2 hours at 4°C with gentle rotation. Pelleted beads were then washed five times with ice-cold RIPA buffer. The immunoprecipitates were eluted from the beads by boiling in sample buffer NuPAGE LDS (Thermo Fisher Scientific) with 200 mM DTT for 5 min at 95°C. The samples were then analyzed by SDS-PAGE and immunoblotting using the indicated antibodies.

### Substrate stretch system

For mechanical stretch experiments, a stretch device (STB-190-XY, Strex Inc.) was used that can apply strain along one or both axes. Polydimethylsiloxane (PDMS) membranes were coated with fibronectin (10 μg/ml; Millipore). HeLa cell monolayers have been cultured on deformable PDMS membranes mechanically linked to actuators that induce strain throughout the surface.

### Mass spectrometry

HeLa cells were lysed in 1% NP-40 lysis buffer (150 mM NaCl, 20 mM Hepes, and 0.5 mM EDTA) containing complete protease and phosphatase inhibitor cocktail (Roche). Total protein lysate (10 mg) was incubated for 3 hours with protein A Dynabeads (Invitrogen) and 10 μg of RASSF1A antibody (Atlas, #HPA040735) or rIgG at 4°C. After the immunoprecipitation, beads were subjected to two additional washes with water before performing an on-bead digest using Smart trypsin (Thermo Fisher Scientific). Smart digest buffer (200 μl) was added to the dry beads and incubated with 4 μl of smart trypsin for 1 hour at 70°C. Trypsin digestion was stopped by adding 1% trifluoroacetic acid (TFA) (final concentration) and trypsin beads removed using the magnet. Tryptic peptides solution was desalted using HRP SOLA cartridges (Thermo Fisher Scientific) and dried down.

Dried tryptic peptides were reconstituted in 15 μl of LC-MS grade water containing 2% acetonitrile and 0.1% TFA. Seven percent of the sample was analyzed by liquid chromatography–tandem mass spectrometry (LC-MS/MS) using a Dionex UltiMate 3000 UPLC coupled to a Q-Exactive mass spectrometer (Thermo Fisher Scientific). Peptides were loaded onto a trap column (PepMap C18; 300 μm × 5 mm, 5 μm particle size, Thermo Fisher Scientific) for 1 min at a flow rate of 20 μl/min before being chromatographically separated on a 50-cm-long Easy-Spray Column (ES803, PepMap C18, 75 μm × 500 mm, 2 μm particle, Thermo Fisher Scientific) with a gradient of 2 to 35% acetonitrile in 0.1% formic acid and 5% DMSO with a 250 nl/min flow rate for 60 min (*77*). The Q-Exactive was operated in a data-dependent acquisition mode to automatically switch between full MS scan and MS/MS acquisition. Survey-full MS scans were acquired in the Orbitrap mass analyzer over a mass/charge ratio (*m*/*z*) window of 380 to 1800 and at a resolution of 70 k (AGC target at 3 × 10^6^ ions). Before MSMS acquisition, the top 15 most intense precursor ions (charge state ≥2) were sequentially isolated in the Quad (*m*/*z* 1.6 window) and fragmented in the HCD collision cell (normalized collision energy of 28%). MS/MS data were obtained in the Orbitrap at a resolution of 17,500 with a maximum acquisition time of 128 ms, an AGC target of 1 × 10^5^, and a dynamic exclusion of 27 s.

#### Data analysis

LC-MS/MS raw files were label-free quantified using the Progenesis QI for proteomics software (v4. Non-linear dynamics, Waters). Briefly, all MS files were automatically aligned to an assigned reference run (control2) to minimize retention time variability between runs, experimental groups were defined, and MS peak picking was automatically performed. For proteomics analysis, a generic .mgf file was generated and searched using MASCOT (v2.3). The data were searched against the Human SwissProt database using a peptide mass tolerance of 10 parts per million, an MS/MS mass tolerance of 0.05, selecting trypsin as an enzyme (1 missed cleavage allowed), and oxidation of methionine, deamidation of NQ, phosphorylation of ST and acetylation of K, as variable modifications. MASCOT data were filtered using an ion score cutoff of 20 and a false discovery rate (FDR) of 1%. Data were then imported to Progenesis QI. Data were normalized to all proteins identified and quantified using the HiN relative quantitation (top N most abundant peptides at 3). Analysis of variance (ANOVA) statistical analysis was applied.

### Mice and tissue preparation

For immunostaining, WT and Rassf1AA133S/A133S male/female mice at 27 to 30 and 18 weeks old were used. The brains were postfixed overnight at 4°C in 4% paraformaldehyde, embedded in paraffin, and sectioned at 5 μm in thickness for immunostaining studies. Sections were rehydrated through graded alcohols to H_2_O and incubated in 1% H_2_O_2_ for 30 min. Nonspecific binding was blocked by incubation with 5% normal goat serum (NGS) for 1 h, which was replaced with D27 antiserum and incubated overnight at 4°C in a saturated humidity chamber.

### Magnetic resonance imaging

MRI images were acquired at 100-μm isotropic resolution on an Agilent 7 T scanner. Data were zero-filled and images were reconstructed at 50 μm isotropic resolution. The MRI images were analyzed with Fiji/ImageJ software using the segmentation editor tool (https://imagej.net/plugins/segmentation-editor). The cortex region was selected at different planes and thresholds were set. The selection was then applied to the entire MRI stack and measured to obtain the total area of the cortex. For measurement of the ventricles, maximum intensity projections of the MRI stack were obtained using Fiji/ImageJ. The ventricle region was selected and the area of the 2D stack was measured using the analyze particle tool.

### Quantifications and statistics

Statistical analysis was performed with the GraphPad Prism software (Version 7.01). For all experiments, statistical analysis was carried out using a Student’s *t* test and statistical significance was defined as *P* < 0.05. Statistical significance is reported in the figures. All data are expressed as mean ± SEM. Fluorescence intensity plots were generated using ImageJ software (NIH). For the quantification of cells with nuclear actin, cells that displayed a diffusely distributed nuclear actin signal were quantified as negative, whereas cells displaying nuclear actin filaments of any type or size were quantified as positive. Quantifications of changes in protein level were calculated using ImageJ software from NIH. To quantify the fluorescence at the NE, cells were costained for Lamin A/C and quantification was performed in ImageJ after background subtraction and thresholding based on the Lamin A/C channel to select the nuclear lamina.
