# Supplementary material for: ATR-hippo drives force signaling to nuclear F-actin and links mechanotransduction to neurological disorders
Source: Sci Adv. 2025 Feb 14;11(7):eadr5683. doi: 10.1126/sciadv.adr5683 (PMC11827640; doi:10.1126/sciadv.adr5683)
Supplement: Supplementary file 1 — Figs. S1 to S7 Tables S1 to S3 [file sciadv.adr5683_sm.pdf]

Science Advances
AAAS

Supplementary Materials for

ATR-hippo drives force signaling to nuclear F-actin and links mechanotransduction to neurological disorders

Maria Chatzifrangkeskou *et al.*

Corresponding author: Eric O'Neill, eric.oneill@oncology.ox.ac.uk;
Maria Chatzifrangkeskou, chatzifrangkeskou.maria@ucy.ac.cy

Sci. Adv. **11**, eadr5683 (2025)
DOI: 10.1126/sciadv.adr5683

This PDF file includes:

Figs. S1 to S7
Tables S1 to S3

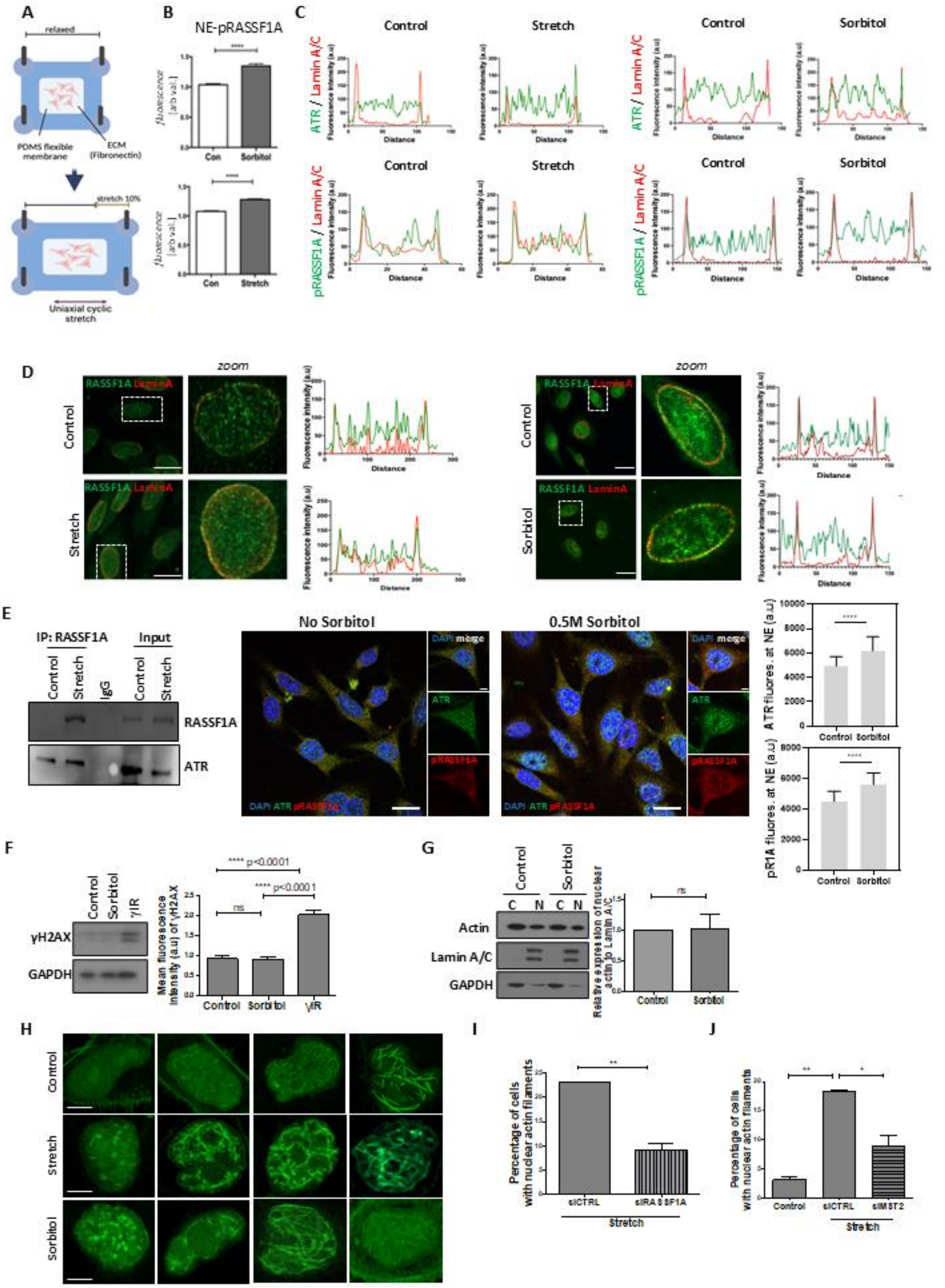

Figure S1: DDR-independent activation of ATR at the NE induces pRASSF1A.

A) Diagrammatic representation of mechanical stretching of the cells for confocal microscopy used to induce 10% uniaxial stretch of HeLa cells. B) Fluorescence intensity of ATR and pRASSF1A at the NE

normalised to nuclear intensity. C) Fluorescence intensity profiles of ATR and pRASSF1A overlap with Lamin A/C across the nuclei of control and sorbitol-treated cells (Fig 1A). D) Images and fluorescence intensity profiles of RASSF1A and Lamin A/C in HeLa cells under mechanical stretch or sorbitol. E) Immunoprecipitation of endogenous RASSF1A showing interaction with ATR in mechanically induced cells. Immunofluorescence images of ATR with pRASSF1A showing co-localization in sorbitol-treated cells. F) Western blot and fluorescence intensity of γH2AX from HeLa cells exposed to sorbitol or 5 Gy ionizing radiation (γIR) as a positive control. G) Western blot analysis of actin in nuclear/cytoplasmic fractionated HeLa cell lysates from control or sorbitol exposed cells. GAPDH as a control for Cytoplasmic (C) or Lamin A/C for nuclear (N) fractions. H) Nuclear actin filament structures observed in control, sorbitol-treated and mechanically induced cells. I) Percentage of cells with nuclear actin filaments in RASSF1A depleted stretched cells. J) Percentage of cells with nuclear actin filaments in MST2 depleted stretched cells. Scale bars, 5 µm. Error bars represent mean ± SEM from two independent experiments.

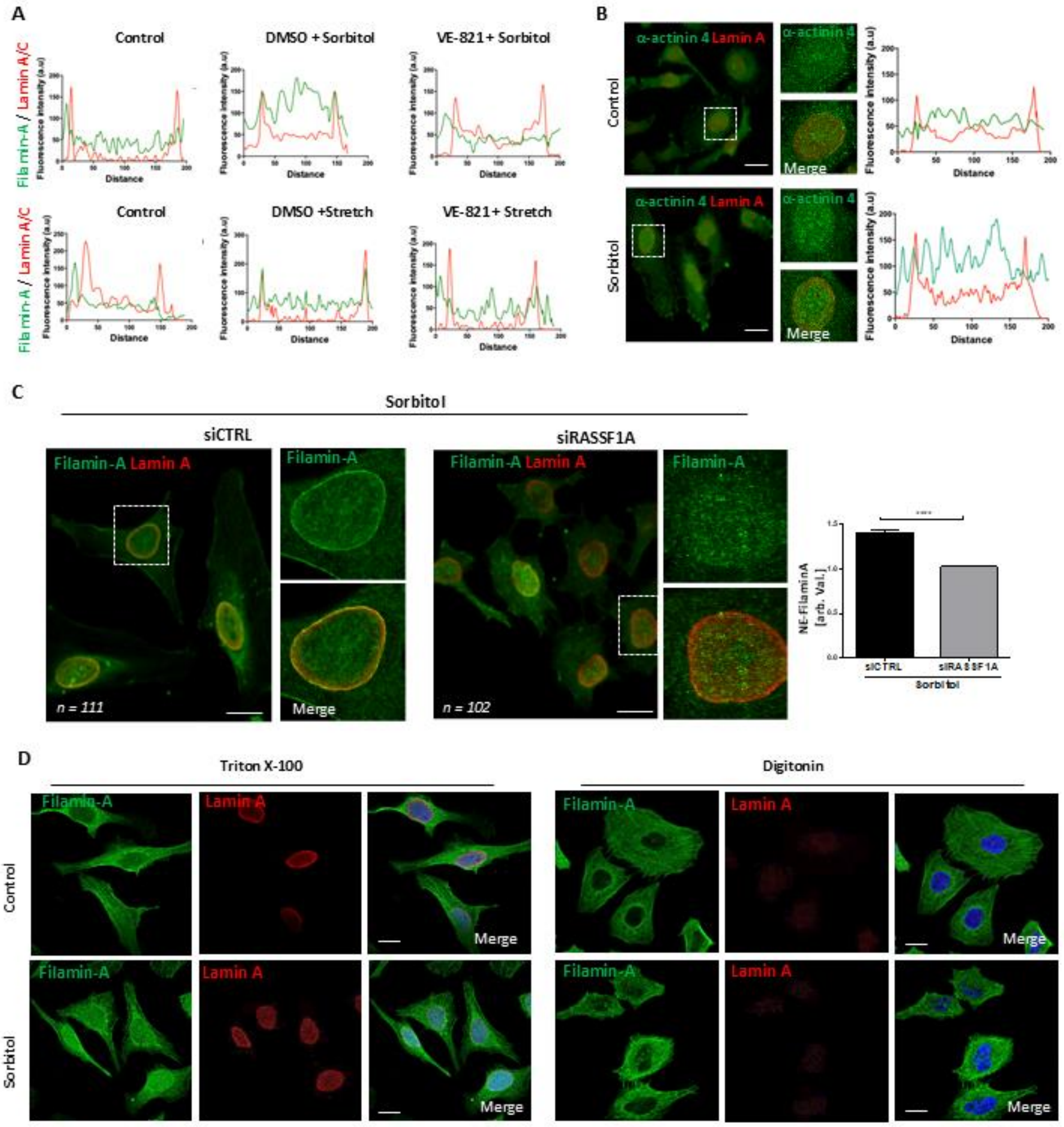

Figure S2: Filamin-A NE localization is dependent on RASSF1A phosphorylation.

A) Fluorescence intensity profiles of Filamin-A overlap with Lamin A/C across the nuclei of control and sorbitol-treated cells (Fig 2D). B) Representative images and fluorescence intensity profiles of α-actinin 4 and Lamin A/C. C) siRNA kncokdown of RASSF1A showing a significant reduction in localisation of Filamin-A from the NE in sorbitol-treated cells. D) Immunofluorescence images of Filamin-A and Lamin A/C in control and sorbitol-treated cells permeabilized with either Triton X-100 or Digitonin. Scale bars, 5 µm. Error bars represent mean ± SEM from two experiments.

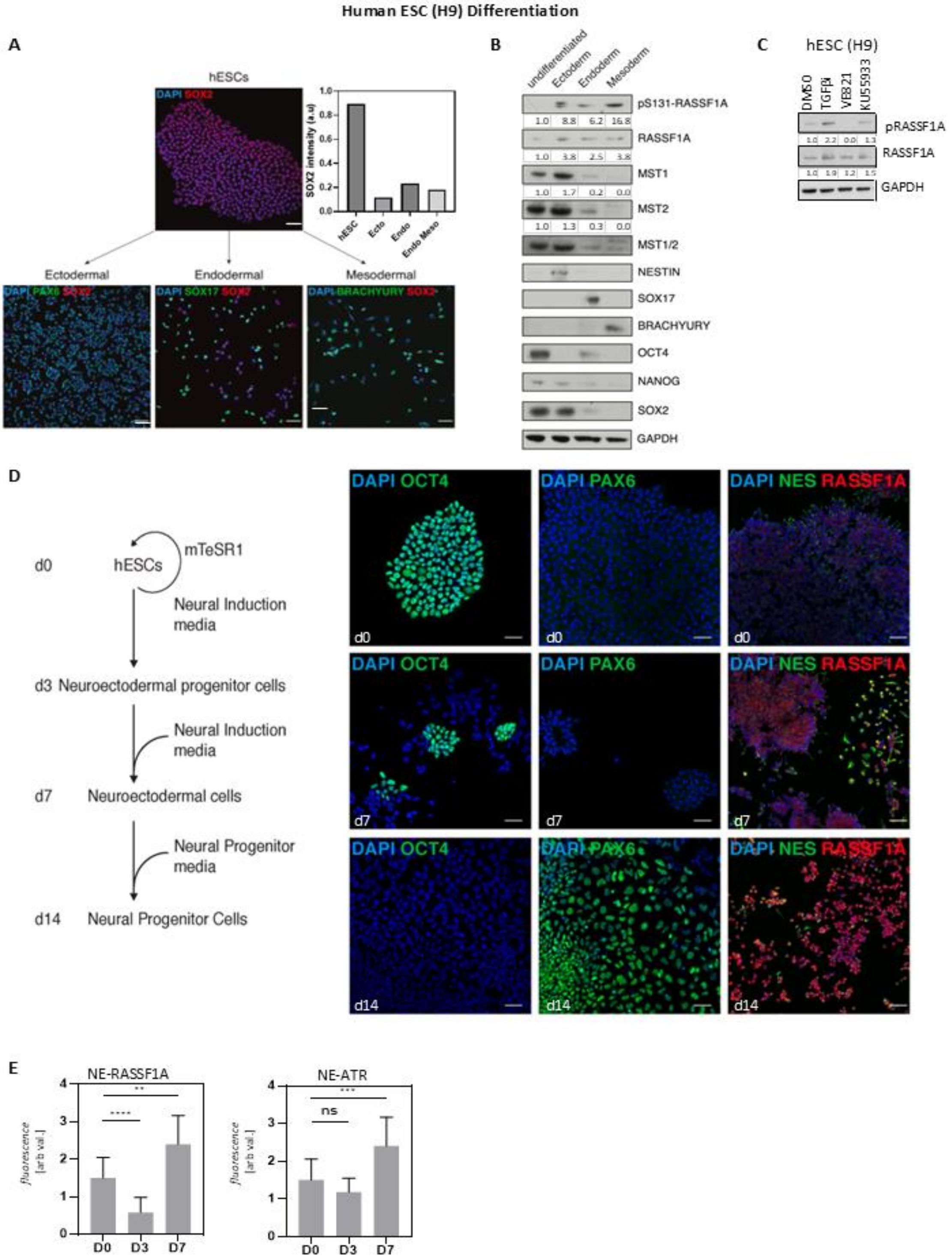

Figure S3: hESC differentiation activates ATR-RASSF1A

A) Immunofluorescence images of H9 human embryonic stem cells (hESCs) in maintenance (mTESR) or differentiation (StemDiff) media for 7 days and stained with the pluripotency marker SOX2 and markers for ectoderm (PAX6), endoderm (SOX17)and mesoderm (BRACHYURY), neuronal progenitor marker PAX6 or RASSF1A. B) Western blot of H9 lysates from (A) with indicated antibodies. Numbers beneath western blots indicate quantification using imageJ. (C) Western blots of H9 hESCs lysates treated with DMSO, TGFbi as a positive control (Pefani, Mol Cell 2016), ATR(VE821) or ATM (Ku55933) inhibition and stained with indicated antibodies. (D) Scheme for differentiating hESC into neuroectodermal progenitor Nestin+ve and neural progenitor cells Nestin+ve PAX6+ve with fluorescent images for OCT4, PAX6, NESTIN (NES) and RASSF1A. E) Quantification of RASSF1A and ATR at the NE of ReNcell VM cells during differentiation.

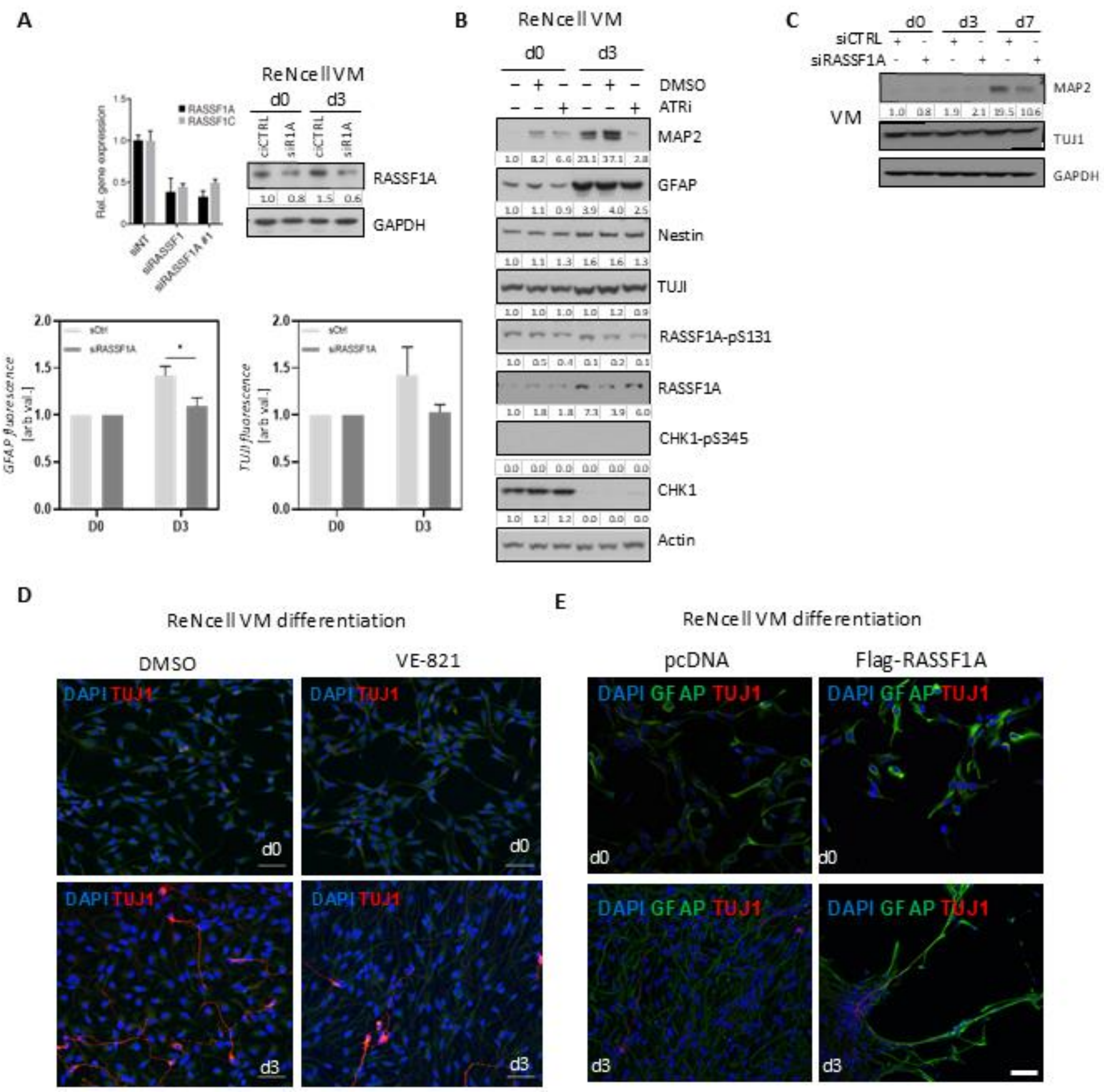

Figure S4: hNSC differentiation requires ATR-RASSF1A

A) ReNcell VM cells nucleofected with siRNA control (siCTRL) or siRASSF1 at day 0 and day 3 post withdrawal of EGF/FGF to initiate differentiation. mRNA and western blots indicate reduced expression. Quantification of GFAP and TUJI fluorescence intensity in siRASSF1A-transfected ReNcell VM cells. Error bars represent mean ± SEM from two experiments. (related to Fig 3E). B) Representative western blots for VM cells differentiated to d3 in the presence of DMSO or ATRi (VE-821) and stained with indicated antibodies. Numbers beneath western blots indicate quantification using imageJ. C) As in B) with siRASSF1 vs siRNA control (siCTRL). D) TUJI+ve staining in differentiating VM cells at d3 in the presence of ATRi (VE821). E) TUJI+ve and GFAP+ve cells in differentiating VM cells with exogenous expression of FLAG-RASSF1A versus control.

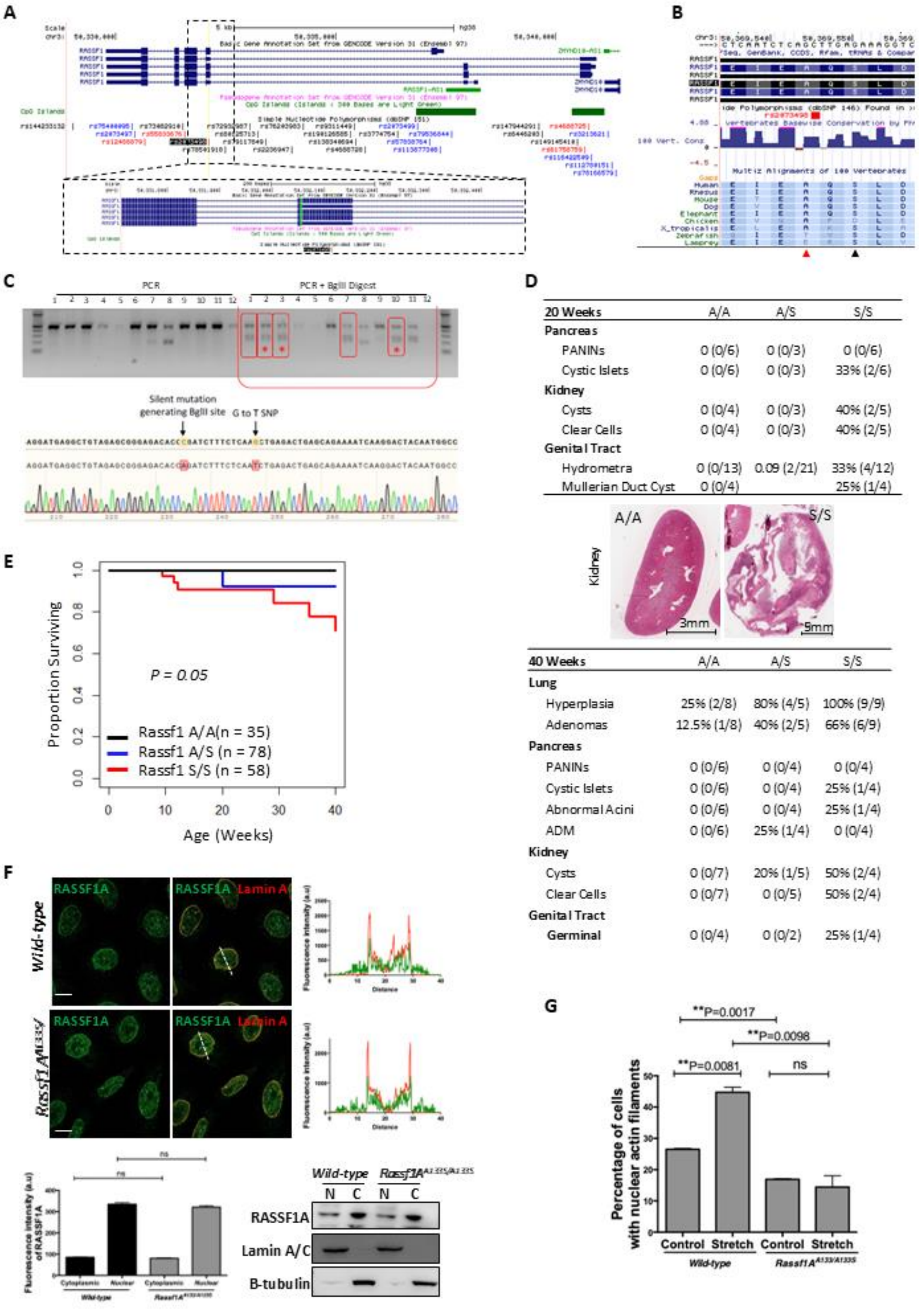

20 Weeks	A/A	A/S	S/S
**Pancreas**			
PANINs	0 (0/6)	0 (0/3)	0 (0/6)
Cystic Islets	0 (0/6)	0 (0/3)	33% (2/6)
**Kidney**			
Cysts	0 (0/4)	0 (0/3)	40% (2/5)
Clear Cells	0 (0/4)	0 (0/3)	40% (2/5)
**Genital Tract**			
Hydrometra	0 (0/13)	0.09 (2/21)	33% (4/12)
Mullerian Duct Cyst	0 (0/4)		25% (1/4)

40 Weeks	A/A	A/S	S/S
**Lung**			
Hyperplasia	25% (2/8)	80% (4/5)	100% (9/9)
Adenomas	12.5% (1/8)	40% (2/5)	66% (6/9)
**Pancreas**			
PANINs	0 (0/6)	0 (0/4)	0 (0/4)
Cystic Islets	0 (0/6)	0 (0/4)	25% (1/4)
Abnormal Acini	0 (0/6)	0 (0/4)	25% (1/4)
ADM	0 (0/6)	25% (1/4)	0 (0/4)
**Kidney**			
Cysts	0 (0/7)	20% (1/5)	50% (2/4)
Clear Cells	0 (0/7)	0 (0/5)	50% (2/4)
**Genital Tract**			
Germinal	0 (0/4)	0 (0/2)	25% (1/4)

Figure S5: **Generation of the rs2073498 SNP Mouse.**

A) Genome browser view of *RASSF1* gene. Common SNPS (MAF > 5%) are shown. Zoomed region showing position of the rs2073498 SNP at exon 3 of the *RASSF1* gene. B) Conservation between human SNP and other vertebrates. The wild-type SNP allele (red arrowhead) and ATM phosphorylation site (black arrowhead) are conserved across most species, including mice. C) Screening of ES cells following CRISPR/Cas9 knock-in. A silent mutation that generated a BglII site was introduced along with the SNP. PCR amplification of the SNP region (left) and BglII digested PCR products (right) are shown. Putative

knock-in clones are shown in red. Starred samples were taken forward for sequencing and sequencing read of successful homozygous knock-in clone. D) Initial pathology of mouse genotypes at 20 week and 40 weeks reveal an increased tendency for cysts and hyperplasia and lead to a significant impact on overall survival (E). F) MEFs from WT and Rassf1A$^{A133S/A133S}$ mice stained for RASSF1A, and fluorescence intensity profiles indicated no difference in RASSF1A association with the NE. Quantification of fluorescence intensity and cell fractionation show no change in the expression of RASSF1A between WT and Rassf1A$^{A133S/A133S}$ cells. G) Percentage of WT and Rassf1A$^{A133S/A133S}$ MEFs with nuclear actin filaments. Scale bars, 10 µm. Error bars represent mean ± SEM from two independent experiments.

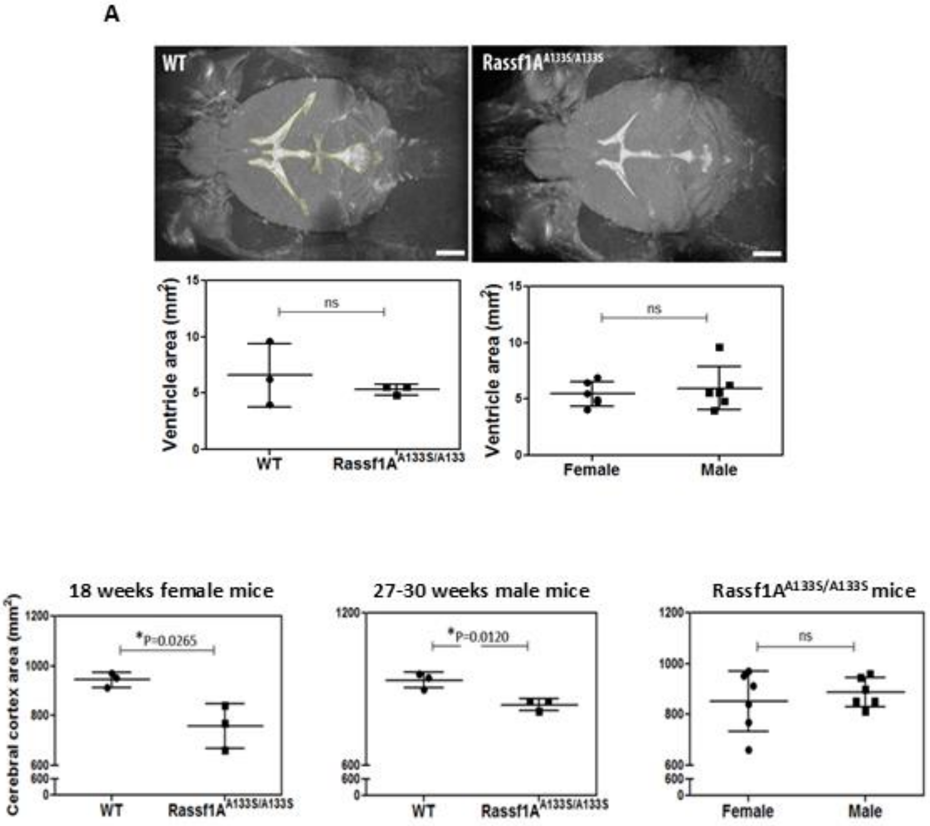

Figure S6: Cortex variation is independent of age and gender.
A) MRI analysis of brain ventricles showed no statistically significant changes between WT and Rassf1AA133S/A133S or between the genders for the Rassf1AA133S/A133S mice. Scale bar, 2 µm. Error bars represent mean ± SD. B) Cortex volumes are independent of age/gender.

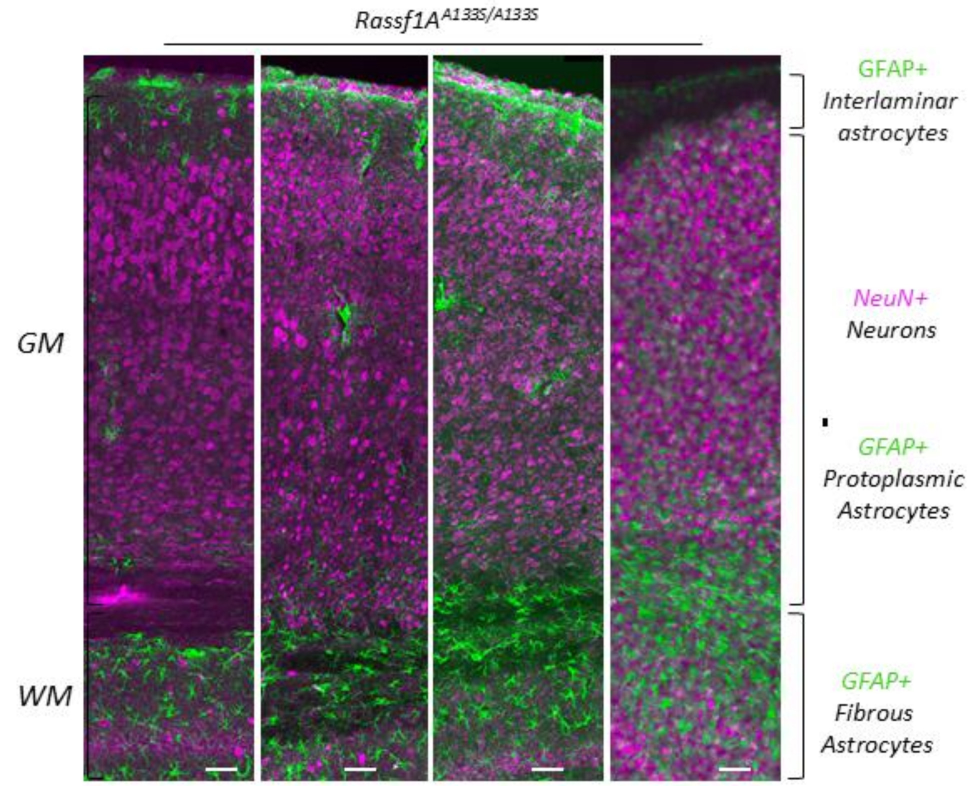

Figure S7: Cortical variations in neuronal cells Rassf1AA133S/A133S mice.
Serial coronal sections of mice brains, 30 µm, (1.75–2.8 mm posterior to bregma) were taken for immunostaining and quantification (Figure 5D). Maximum intensity projections of confocal images, 200 µm sections of the brain, highlighting the PTLp regions, showing the various different GFAP+ (green) astrocytes and NeuN+ neurons (magenta) in the GM and WM. Scale bar 100 µm.

Protein	Control	Sorbitol	Fold change
Filamin-B	63	1366	20.67460317
Spectrin beta chain, non-erythrocytic 1	70	1216	16.49640288
Pyruvate kinase PKM	62	574	8.258064516
Filamin-A	321	2808	7.747663551
LIM domain and actin-binding protein 1	228	1644	6.224175824
60 kDa heat shock protein, mitochondrial	81	548	5.765432099
Alpha-actinin-1	251	1444	4.76247505
Alpha-enolase	68	389	4.713235294
Serine/arginine repetitive matrix protein 2	53	274	4.219047619
Golgin subfamily A member 3	116	596	4.13362069
Leucine zipper protein 1	71	364	4.126760563
Alpha-actinin-2	147	652	3.431972789
Protein phosphatase 1 regulatory subunit 12A	133	553	3.173584906
Alpha-actinin-4	578	2255	2.900519031
Alpha-actinin-3	123	453	2.678861789
Mitotic interactor and substrate of PLK1	155	569	2.670967742
Neurabin-2	131	452	2.459770115
Heat shock protein HSP 90-alpha	80	276	2.45
Unconventional myosin-Ie	160	547	2.41875
Heat shock protein HSP 90-beta	129	424	2.299610895
Unconventional myosin-VI	74	243	2.283783784
Dedicator of cytokinesis protein 7	82	259	2.158536585
EF-hand domain-containing protein D2	79	245	2.114649682
Endoplasmic reticulum chaperone BiP	227	702	2.099337748
POTE ankyrin domain family member I	439	1343	2.059225513
POTE ankyrin domain family member J	426	1284	2.017626322
Putative beta-actin-like protein 3	471	1410	1.996811902
116 kDa U5 small nuclear ribonucleoprotein component	38	112	1.934210526
Glyceraldehyde-3-phosphate dehydrogenase	78	221	1.84516129
14-3-3 protein theta	67	181	1.721804511
Polypyrimidine tract-binding protein 1	56	149	1.660714286
Stress-70 protein, mitochondrial	235	617	1.623404255
Heat shock protein HSP 90-alpha A2	55	144	1.618181818
Drebrin	344	897	1.60755814
60S acidic ribosomal protein P0	50	128	1.585858586
Heterogeneous nuclear ribonucleoprotein K	120	310	1.583333333
Protein flightless-1 homolog	225	542	1.406666667
Serine/threonine-protein phosphatase PP1-gamma catalytic subunit	62	149	1.403225806
Prelamin-A/C	174	418	1.399425287
Leucine-rich repeat flightless-interacting protein 2	135	323	1.397769517

Table S1: Mass Spectrometry of RASSF1A immunoprecipitates. Endogenous immunoprecitiates of RASSF1A with associated proteins that were enriched in the presence of 0.5M sorbitol.

Name	Protein Score
POTE ankyrin domain family member E	1670
Spectrin alpha chain, non-erythrocytic 1	1346
Dystonin	333
Sperm-specific antigen 2	319
Unconventional myosin-X	236
Tubulin beta-6 chain	231
Tubulin beta-8 chain	223
Tight junction protein ZO-1	168
Serologically defined colon cancer antigen 8	134
Caspase-8	132
Transketolase	127
L-lactate dehydrogenase A chain	124
Signal recognition particle 14 kDa protein	121
ATP-dependent zinc metalloprotease YME1L1	120
RNA transcription, translation and transport factor protein	114
Taperin	112
14-3-3 protein epsilon	112
Fructose-bisphosphate aldolase A	111
Beta-enolase	110
Luc7-like protein 3	110
14-3-3 protein zeta/delta	103
60S acidic ribosomal protein P0-like	102
Leucine-rich repeat flightless-interacting protein 1	98
Poly(rC)-binding protein 2	97
Peptidyl-prolyl cis-trans isomerase B	96
Thioredoxin	96
Cleavage and polyadenylation specificity factor subunit 2	96
Putative RNA-binding protein Luc7-like 2	96
Protein enabled homolog	96
40S ribosomal protein S5	95
Poly(rC)-binding protein 1	95
T-complex protein 1 subunit epsilon	91
Cleavage and polyadenylation specificity factor subunit 1	89
Fragile X mental retardation syndrome-related protein 1	89
Ras GTPase-activating-like protein IQGAP2	89
T-complex protein 1 subunit alpha	88
ELAV-like protein 1	87
DNA replication licensing factor MCM3	84
10 kDa heat shock protein, mitochondrial	82
Synaptopodin	80
Heat shock protein 105 kDa	80
Protein S100-A10	76
Phosphate carrier protein, mitochondrial	76
T-complex protein 1 subunit theta	76
KH domain-containing, RNA-binding, signal transduction-as	76
Protein MB21D2	75
Inosine-5'-monophosphate dehydrogenase 2	75
Phostensin	75
ATPase family AAA domain-containing protein 3B	72
ATPase family AAA domain-containing protein 3C	71
Scaffold attachment factor B2	71
Abscission/NoCut checkpoint regulator	70
60S ribosomal protein L5	70
Peroxiredoxin-6	68
Single-stranded DNA-binding protein 2	67
Vam6/Vps39-like protein	67
Profilin-1	66
Src substrate cortactin	65
Cystatin-B	65
RuvB-like 1	64
Charged multivesicular body protein 4b	63
Pyruvate kinase PKLR	62
WD repeat-containing protein 11	61
L-lactate dehydrogenase B chain	60
Poly(rC)-binding protein 3	60
Actin-related protein 2/3 complex subunit 1B	60
Importin subunit alpha-1	59
60S ribosomal protein L27	59
60S ribosomal protein L9	59
Nucleoside diphosphate kinase B	58
Splicing factor, proline- and glutamine-rich	57
Tumor suppressor ARF	56

Ubiquitin carboxyl-terminal hydrolase 7	55
Elongation factor 1-delta	55
Pleckstrin homology domain-containing family G member 3	54
Serine/arginine-rich splicing factor 1	53
40S ribosomal protein S10	53
Histone deacetylase 1	53
Plasminogen activator inhibitor 1 RNA-binding protein	52
Protein-L-isoaspartate(D-aspartate) O-methyltransferase	52
THAP domain-containing protein 1	51
Mucolipin-2	50
RNA-binding protein 8A	49
Fascin	49
Splicing factor U2AF 65 kDa subunit	48
Polyadenylate-binding protein 5	48
RNA-binding protein 39	46
Piwi-like protein 3	46
C-Jun-amino-terminal kinase-interacting protein 3	45
Cysteine and glycine-rich protein 2	43
40S ribosomal protein S7	43
Transformer-2 protein homolog alpha	43
Reticulocalbin-2	41
ATP synthase subunit O, mitochondrial	40
U4/U6.U5 tri-snRNP-associated protein 2	40
THO complex subunit 4	38
Protein RCC2	36
Ubiquitin carboxyl-terminal hydrolase 7	55
Elongation factor 1-delta	55
Pleckstrin homology domain-containing family G member 3	54
Serine/arginine-rich splicing factor 1	53
40S ribosomal protein S10	53
Histone deacetylase 1	53
Plasminogen activator inhibitor 1 RNA-binding protein	52
Protein-L-isoaspartate(D-aspartate) O-methyltransferase	52
THAP domain-containing protein 1	51
Mucolipin-2	50
RNA-binding protein 8A	49
Fascin	49
Splicing factor U2AF 65 kDa subunit	48
Polyadenylate-binding protein 5	48
RNA-binding protein 39	46
Piwi-like protein 3	46
C-Jun-amino-terminal kinase-interacting protein 3	45
Cysteine and glycine-rich protein 2	43
40S ribosomal protein S7	43
Transformer-2 protein homolog alpha	43
Reticulocalbin-2	41
ATP synthase subunit O, mitochondrial	40
U4/U6.U5 tri-snRNP-associated protein 2	40
THO complex subunit 4	38
Protein RCC2	36

Table S2: Protein associations of RASSF1A that were uniquely identified in the presence of 0.5M sorbitol.

Enigma_thickness

Region	BETA	BETA_SE	P	FDR
bankssts	0	0.0015	0.9874	0.9991
caudalanteriorcingulate	-0.0005	0.0024	0.8448	0.9991
caudalmiddlefrontal	0.0013	0.0012	0.2767	0.99078
cuneus	0.0009	0.0013	0.4967	0.99078
entorhinal	0.0014	0.0038	0.7033	0.99078
frontalpole	-0.0017	0.0027	0.5233	0.99078
Full	0.0008	0.0012	0.4851	0.99078
fusiform	-0.0003	0.0013	0.804	0.9991
inferiorparietal	-0.0005	0.001	0.6455	0.99078
inferiortemporal	-0.0001	0.0015	0.9321	0.9991
insula	-0.0015	0.0015	0.3123	0.99078
isthmuscingulate	-0.0017	0.0021	0.4028	0.99078
lateraloccipital	0.0007	0.0011	0.5581	0.99078
lateralorbitofrontal	0	0.0015	0.9958	0.9991
lingual	0.0007	0.0012	0.5516	0.99078
medialorbitofrontal	-0.0002	0.0016	0.8984	0.9991
middletemporal	0.0003	0.0014	0.8327	0.9991
paracentral	0.0006	0.0014	0.6422	0.99078
parahippocampal	0	0.0032	0.9991	0.9991
parsopercularis	-0.0011	0.0013	0.3715	0.99078
parsorbitalis	0	0.0019	0.9895	0.9991
parstriangularis	0	0.0013	0.9725	0.9991
pericalcarine	-0.0007	0.0013	0.6049	0.99078
postcentral	0.0011	0.0011	0.3109	0.99078
posteriorcingulate	-0.0016	0.0015	0.2857	0.99078
precentral	-0.0005	0.0012	0.6949	0.99078
**precuneus**	**-0.0024**	**0.001**	**0.02091**	**0.73185**
rostralanteriorcingulate	0.0009	0.0022	0.6689	0.99078
rostralmiddlefrontal	0.0005	0.0011	0.6802	0.99078
superiorfrontal	-0.0009	0.0011	0.4076	0.99078
superiorparietal	-0.0006	0.001	0.5481	0.99078
superiortemporal	0.0015	0.0014	0.2804	0.99078
supramarginal	0.0004	0.001	0.7077	0.99078
temporalpole	-0.0034	0.0033	0.3047	0.99078
transversetemporal	0.0012	0.0021	0.5737	0.99078

Table S3: Cortex variation by region ENGIMA
